# A pictorial essay on radiological changes after stereotactic body radiation therapy for lung tumors

**DOI:** 10.1007/s11604-022-01252-7

**Published:** 2022-02-20

**Authors:** Yasuo Matsumoto

**Affiliations:** Department of Radiation Oncology, Niigata Cancer Center Hospital, 2-15-3, Kawagishi-cho, Chuo-ku, Niigata, 951-8566 Japan

**Keywords:** Stereotactic body radiation therapy, Radiological changes, Lung cancer, Radiation pneumonitis

## Abstract

Stereotactic body radiation therapy (SBRT) is a frequently used modality for the treatment of early stage non-small cell lung cancer and oligometastatic disease of the lung. The radiological changes observed in the lung after SBRT are likely to differ from those observed after conventional thoracic radiation therapy, primarily due to the small size of the target volume and highly conformal dose distributions with steep dose gradients from the target to surrounding normal lung tissues used in SBRT. Knowledge of the radiological changes that can occur after SBRT is required to correctly diagnose local failure. Herein, I report several radiological changes specific to SBRT that have been observed.

## Introduction

In the past, radiation therapy for lung tumors consisted primarily of simple two-dimensional irradiation, such as two-port radiation in opposite directions, but advances in technology have made three-dimensional treatment possible. Furthermore, as in the case of image-guided radiotherapy, the position of various organs (especially bones) and target tumors can be confirmed on the treatment table, allowing treatment to be more localized to the tumor. This has made it possible to reduce the dose to the surrounding normal organs and deliver a large dose to the target tumor, resulting in a high control rate [1–4]. Stereotactic body radiotherapy (SBRT) is a treatment method in which a small amount of radiation is precisely focused from multiple directions to deliver an intense dose of radiation to the lesion while minimizing the dose to surrounding normal tissue. Due to the convergence of radiation beams in SBRT, it is common for the planning target volume (PTV) to be smaller than that used for conventional radiation therapy. To concentrate radiation beams from many directions, the slope of the dose distribution around the tumor is steep. Almost all patients are treated with high-dose fractions; therefore, fewer fractions are administered and the treatment period is shorter than those of conventional radiation therapy. For the above reasons, a variety of unique radiological imaging changes after SBRT that are not observed in conventional radiation therapy are seen in a considerable number of cases. We started using SBRT in July 2005 at Niigata Cancer Center Hospital, and the number of cases in which SBRT was used to treat pulmonary tumors (primary lung cancers and metastatic pulmonary tumors) reached 2300 in December 2021. We here present our cases which can be misdiagnosed without careful attention or adequate knowledge on images after SBRT.

## Pulmonary mass-like changes after SBRT

After SBRT, lung tumors gradually decrease in size, but before they completely disappear, the shrinking tumors are usually replaced by radiation pneumonitis. Therefore, evaluation of such tumors after SBRT is difficult in many cases. Radiation pneumonitis gradually involves the surrounding lung tissues and appears as a linear and/or funicular change or high-dense consolidation. In a considerable number of cases, increased density appears after SBRT that resembles primary lung cancers, even though the tumors had actually disappeared [5–14]. This is the most frequent imaging change observed after SBRT. It is very difficult to differentiate between post-SBRT changes and recurrence, and various studies have been conducted to correctly diagnose such CT changes [5, 10, 15–17].

**Fig. 1:**
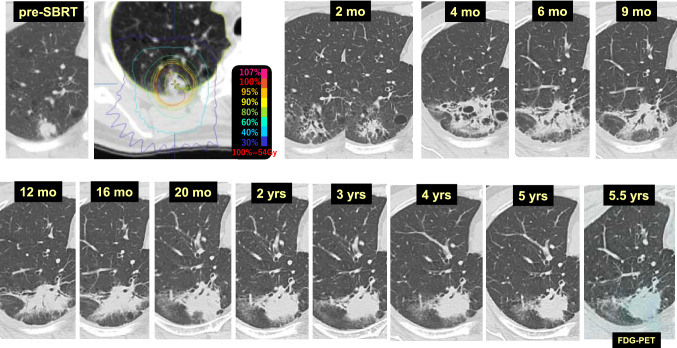
“Primary lung cancer”-like changes after SBRT

Case 1 (Fig. 1) is a lung tumor (histologically unconfirmed) in the right upper lobe (S2) of a 69-year-old male patient. Two months after 54 Gy/4 fr SBRT, tumor reduction and radiation pneumonitis occurring circumferentially were observed synchronously. At 4 months, dense radiation pneumonitis was observed. At 6 months, the radiation pneumonitis showed resorption and convergence, and was shaped like a tumor-like consolidation. Beginning 2 years after SBRT, no changes in the size or shape of the radiation pneumonitis were observed. A fluorodeoxyglucose-positron emission tomography (FDG-PET) scan at 5.5 years revealed no evidence of cancer recurrence, but the radiation fibrosis looked exactly like a primary lung cancer.

## Difference in radiosensitivity in the lung

It is generally thought that the irradiated lung volume and radiation dose are closely associated with the severity of radiation pneumonitis [18, 19]. The V20 index (the lung volume receiving ≥ 20 Gy) is frequently used for radiation therapy to lung lesions [20]. When SBRT can be performed to a limited lung area, it is possible to synchronously irradiate two or more tumors. Depending upon the conditions of a patient, SBRT is given to two or more tumors in the ipsilateral lung or to each lung synchronously.

### Difference in radiosensitivity between lungs

When SBRT to pulmonary metastases in each lung was performed synchronously, we experienced several cases in which the timing and grade of radiation pneumonitis greatly differed between the right and left lungs [11, 12].

**Fig. 2:**
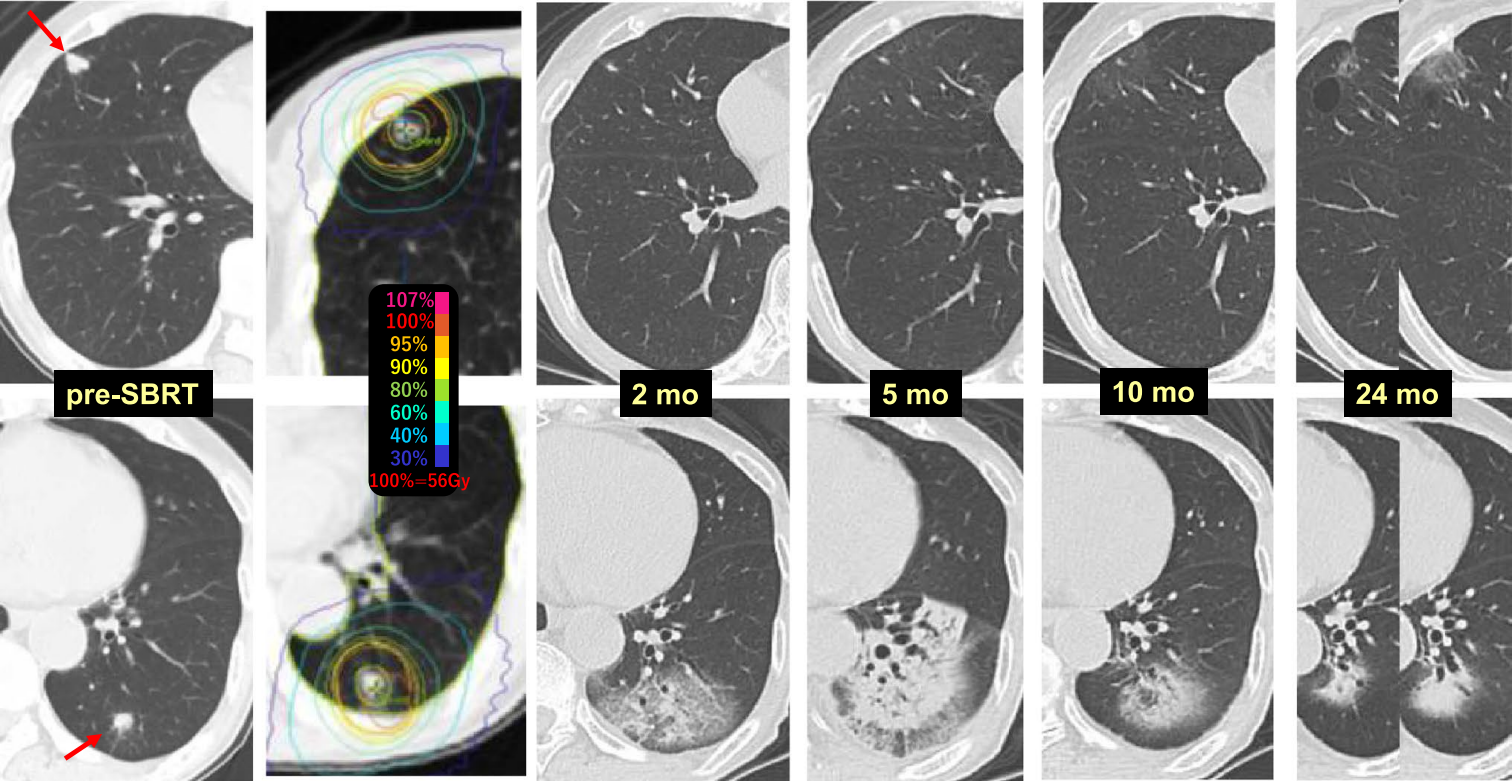
Difference in radiosensitivity between lungs

Case 2 (Fig. 2) involves pulmonary metastases (red arrows) from renal cell carcinoma in each lung of a 76-year-old female. Fifty-six Gy/4 fr SBRT (each PTV was almost equal and the same radiation dose was used) to the two pulmonary metastases was performed. Radiation pneumonitis had appeared in the left lower lung lobe (S10) at 2 months (lower row), and while the tumor in the right upper lobe (S3) was obviously reduced, the SBRT target area showed no pulmonary changes. At 24 months, only a faint pulmonary change was observed in the right upper lobe.

As observed in this case, SBRT performed for pulmonary metastases in both lungs synchronously revealed that the lungs had differing susceptibility to radiation.

### Difference in radiosensitivity between pulmonary lobes

In some cases in which SBRT was administered to tumors near the interlobar pleura, radiation pneumonitis occurs in another pulmonary lobe adjacent to the target lobe, despite the lower dose given to that area, rather than in the target lobe. In addition, later appearance of radiation pneumonitis in lobes adjacent to the target lobe has also been observed.

**Fig. 3:**
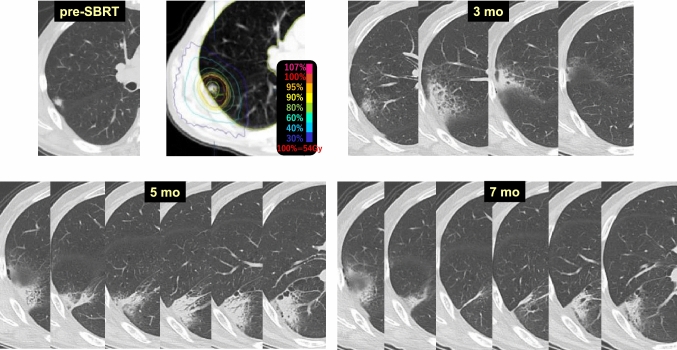
Difference in radiosensitivity between pulmonary lobes

Case 3 (Fig. 3) is a 67-year-old male patient who received SBRT 54 Gy/4 fr to a lung tumor (histologically unconfirmed) in the right upper lobe (S2). At 3 months after SBRT, radiation pneumonitis had appeared in the target area. No imaging changes were observed in the right lower lobe adjacent to the target lobe at this time. At 5 months, radiation pneumonitis was observed in the lower lobe adjacent to the target lobe. At 7 months, both locations of radiation pneumonitis showed signs of resorption. This case illustrates a difference in the timing of the appearance of radiation pneumonitis between different lobes in the ipsilateral lung [11–13].

### Radiosensitivity differences within a pulmonary lobe

Radiosensitivity differences also occur within the same pulmonary lobe: radiation pneumonitis frequently appears earlier in regions that receive a lower dose of radiation compared with that given to the target area [11–13].

**Fig. 4:**
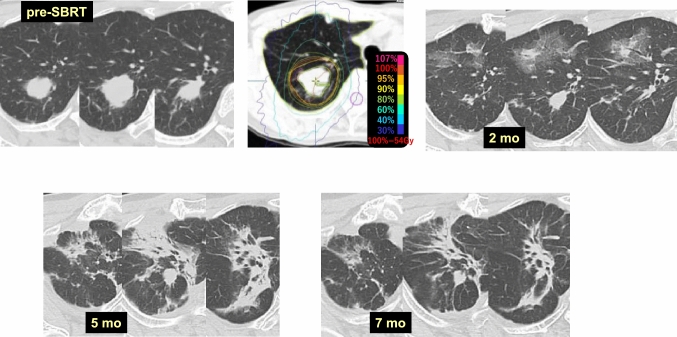
Difference in radiosensitivity within pulmonary lobes (1)

Case 4 (Fig. 4) is a 71-year-old woman who received 54 Gy/4 fr SBRT to a lung tumor (Class V, NOS) in the right upper lobe (S2). At 2 months after SBRT, the tumor showed obvious shrinkage, while radiation pneumonitis was not observed in the surrounding area. In contrast, radiation pneumonitis appeared in the anterior lung area, which obviously received a lower dose of radiation. No radiation pneumonitis had appeared in the target volume at 7 months.

**Fig. 5:**
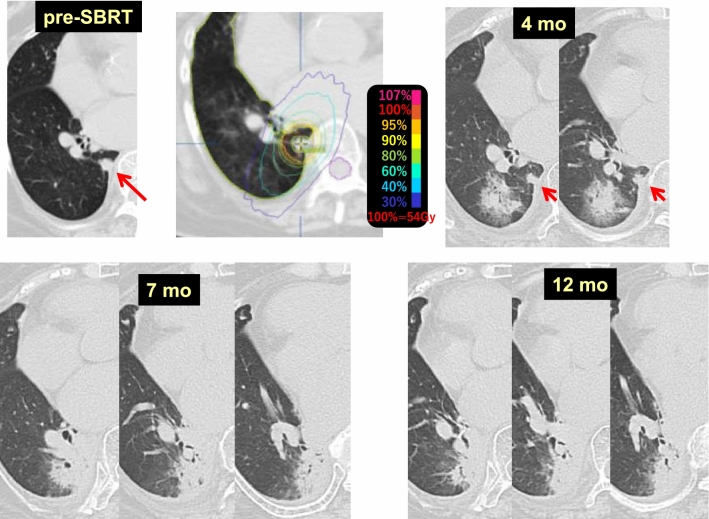
Difference in radiosensitivity within pulmonary lobes (2)

Case 5 (Fig. 5) is an 80-year-old female who received 54 Gy/4 fr SBRT to a lung tumor (histologically unconfirmed) in the lower lobe (S7). At 4 months after SBRT, no radiation pneumonitis was observed in the target area. However, obvious radiation pneumonitis has appeared in a nearby area that had received a lower dose. At 7 months, radiation pneumonitis had also appeared in the target volume.

These cases demonstrate that the timing and grade of radiation pneumonitis vary within different lung areas, even within the same pulmonary lobe. Moreover, we should pay attention to the phenomenon in which lung fields that receive lower radiation doses can cause radiation pneumonitis earlier than the target areas irradiated with higher doses. These observations imply no correlation exists between radiation dose and severity of radiation pneumonitis. Differentiation from organizing pneumonia after radiotherapy can occur in lung regions outside the field of irradiation [21–23]. Murai et al. [24] reported that all patients with organizing pneumonia experienced symptoms of shortness of breath. Unlike radiation pneumonitis, organizing pneumonia rarely results in fibrosis. From this point of view, we believe that organizing pneumonia can be ruled out in this group of patients.

## Enlargement of radiation pneumonitis/fibrosis after SBRT

Radiation pneumonitis changes into fibrosis with time. During the course of fibrosis, the lesion may show temporary enlargement, which can be misdiagnosed as cancer recurrence [6, 7, 25, 26]. As explained above, radiation pneumonitis after SBRT occurs both within and outside the target area at various times. As radiation pneumonitis occurs in different areas surrounding the target, it continuously progresses to fibrosis via traction, conversion, and conglomeration [11, 12]. Enlargement is temporary, as this process involves convergence and shrinkage over time. Therefore, constantly changing radiation pneumonitis and fibrosis are considered to be one mechanism of enlargement of consolidations after SBRT. This phenomenon can result in the delayed changes described above. When a relapse is suspected based on enlargement of consolidations after SBRT, FDG-PET imaging is effective for evaluation, although both false-negative and false-positive FDG uptake must always be considered.

**Fig. 6:**
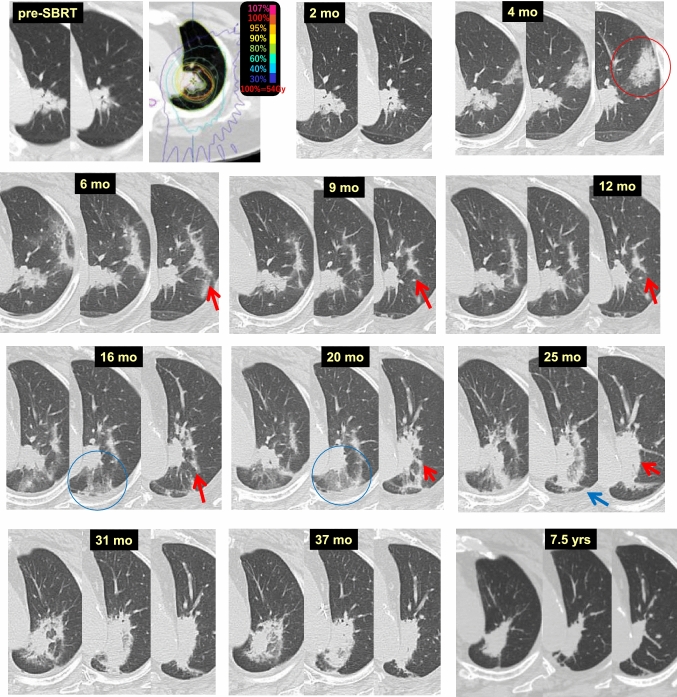
Enlargement of radiation pneumonitis/fibrosis after SBRT (1)

Case 6 (Fig. 6) is a 66-year-old women who received 54 Gy/4 fr SBRT to a lung tumor (adenocarcinoma) in the left upper lobe (S1 + 2). At 2 months after SBRT, the tumor remained unchanged. At 4 months, radiation pneumonitis was observed in a lower dose area (circled in red) far from the target, which appeared almost unchanged. At 6, 9, and 12 months, the pneumonitis in the remote area approached the target area near the hilum of the lung (red arrow). At 16 months, a new, slight increase in density was observed in the dorsal portion of the target area (circled in blue), which was considered to indicate pneumonitis that appeared at a different time. The size of the later radiation pneumonitis had increased at 20 months (circled in blue). At 25 months, the latter pneumonitis was connected to the consolidation of the target (blue arrow). Simultaneously, the former pneumonitis that was gradually approaching had attached to the consolidation of the target (red arrow). At 31 months, the two pneumonitis lesions were separately connected to the target consolidation, which had become enlarged. This process resembled tumor regrowth on imaging. At 37 months, the consolidation was reduced, and this reduction was more obvious at 7.5 years; no recurrence had occurred.

Thus, another mechanism for temporally enlarging consolidations after SBRT is the sequential appearance of pneumonitis and fibrosis to form a larger consolidation [11–14].

**Fig. 7:**
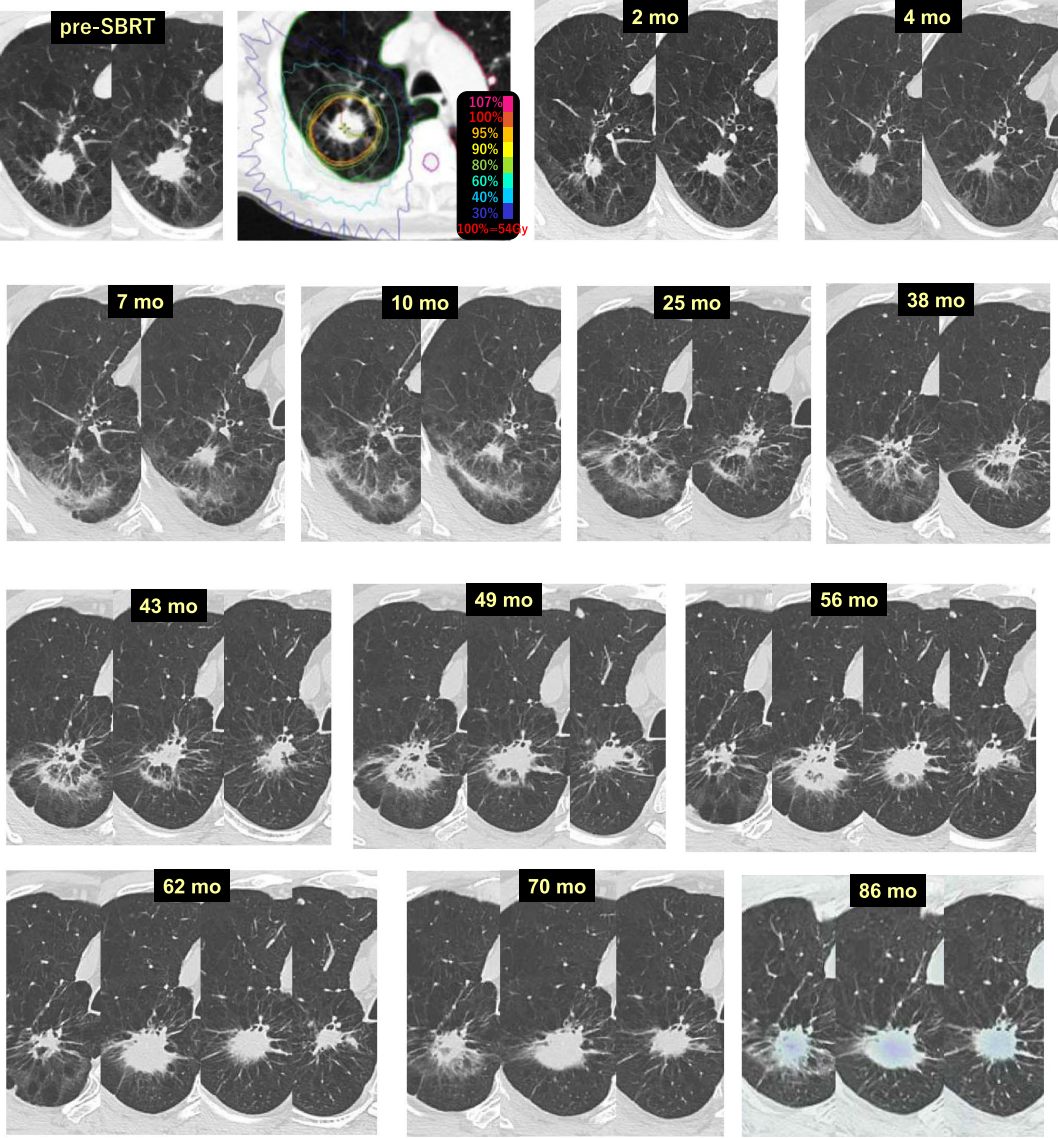
Enlargement of radiation pneumonitis/fibrosis after SBRT (2)

Case 7 (Fig. 7) is a 71-year-old male who received 54 Gy/4 fr SBRT for a lung tumor (adenocarcinoma) in the right upper lobe (S1). At 2 months after SBRT, the tumor was reduced in size due to SBRT and continued to decrease in volume. At 7 months, radiation pneumonitis appeared in the dorsal side of the target area, and it gradually extended to the area of the former tumor. Although no remarkable changes were observed at 43 months after SBRT, at 49 months, the density of the lung area close to the tumor had increased, and the air density in the consolidation had disappeared at 62 months, suspicious of tumor recurrence. After that, no changes in the consolidation were observed at 70 months, and relapse was ruled out by FDG-PET at 86 months.

## Can radiation pneumonitis occur twice?

It is generally believed that radiation pneumonitis will not occur twice. However, second, and rarely, third, instances of radiation pneumonitis appear in many cases in the same lung and even in the same lobe, but are overlooked. This phenomenon represents reverse of that described in  “Radiosensitivity differences within a pulmonary lobe”. After radiation pneumonitis appears in and around the PTV, pneumonitis in normal lung tissue near the target that received a much lower dose of radiation appears later. CT may reveal increased density that resembles pneumonia near or slightly distant from the PTV in the absence of acute inflammation. Unlike typical pneumonia, radiation pneumonitis almost always progresses to fibrosis [11, 12]. Some of these cases may be diagnosed as tumor recurrences.

**Fig. 8:**
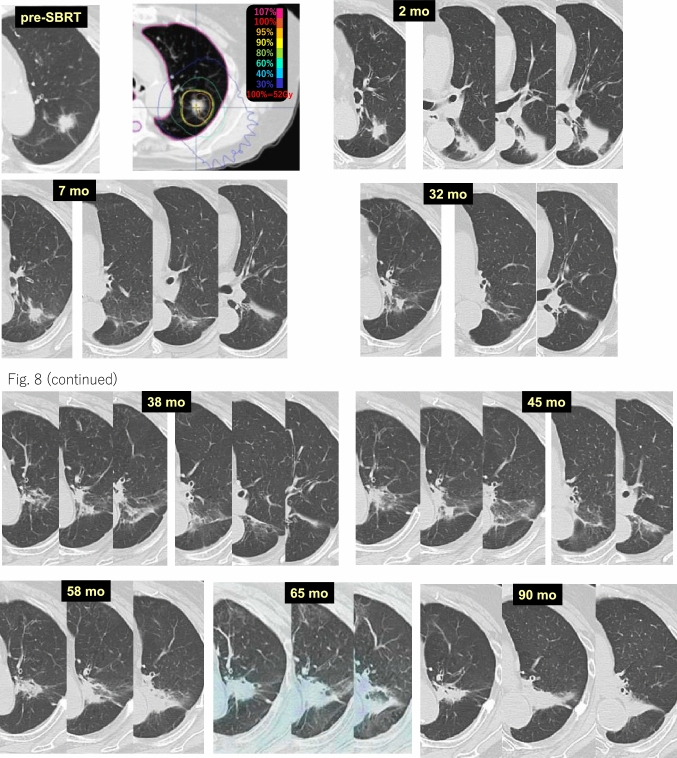
Can radiation pneumonitis occur twice?

Case 8 (Fig. 8) is an 81-year-old female who received 52 Gy/4 fr SBRT for a lung tumor (histologically unconfirmed) in the left upper lobe (S1 + 2). At 2 months after SBRT, radiation pneumonitis appeared in the left lower lobe (S6) next to S1 + 2, while no pneumonitis was observed in the target area. Radiation pneumonitis in the target area did not appear for a long time. However, at 58 months after SBRT, the depth of the density of the target area increased. At 65 months, no abnormal accumulation of density appeared on FDG-PET. At 90 months, unlike common inflammation, fibrotic consolidation remained.

## Delayed radiation pulmonary damage

As with conventional radiation therapy, radiation pneumonitis appears within 6 months in many patients after SBRT, while imaging changes indicating radiation pneumonitis surrounding and in the target area sometimes appear more than 3 years later [12, 14] (Fig. 8). According to Dahele et al. [5], acute CT changes are observed in about half of patients, while late CT changes are seen in almost all patients, with the notable exception of 25% of SBRT patients who do not experience their first CT change until more than 1 year later. The CT changes are dynamic, and in about half of patients, the changes continue for more than 2 years. One hypothesis suggests that SBRT-induced bronchial stenosis and obstruction cause these delayed pulmonary changes, because they occur over a long duration. Bronchial stenosis and obstruction are often observed after a few or several years in cases in which the major bronchi were irradiated with high-dose radiation [13, 14]. If the peripheral side of the subsegmental bronchi are irradiated with high-dose radiation, they become stenotic and obstructed much earlier than the wider major bronchi. We experienced a case in which subsegmental bronchial cancer could not be observed with bronchoscopy 2 months after SBRT due to stenosis of the bronchus [13]. The narrow bronchi of regions that receive a lower dose of radiation gradually become stenotic over time; however, the density of delayed pulmonary changes is radiologically pneumonia-like and not accompanied by a reduction in lung volume, and sometimes, air remains in the bronchi in the region. Therefore, atelectasis by stenosis and/or obstruction of the bronchi is likely not the primary cause [12–14]. Based on these finding, late radiographical imaging changes may be called “delayed radiation pneumonitis.” Delayed imaging changes tend to occur in patients with pulmonary emphysema. Individuals with moderate-to-severe pulmonary emphysema occasionally have no radiation pneumonitis. However, they usually experience radiological changes indicating radiation pneumonitis or tumor-like consolidations over time. Depending on the lesions that form after SBRT, they may be diagnosed as recurrences; therefore, it is necessary to continue close follow-up.

**Fig. 9:**
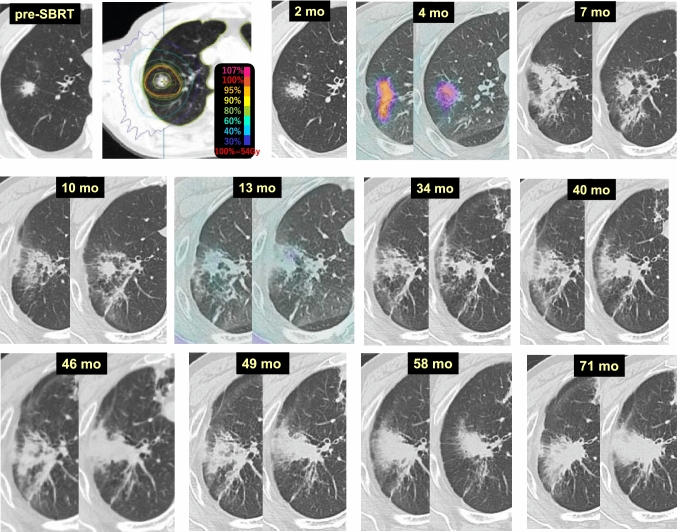
Delayed pulmonary damage (1)

Case 9 (Fig. 9) is an 81-year-old male who received 54 Gy/4 fr SBRT to a lung tumor (adenocarcinoma) in the right upper lobe (S1). At 2 months after SBRT, the tumor edge became obscure. FDG-PET after 4 months revealed FDG accumulation indicating radiation pneumonitis. Although the tumor density was barely reduced at 7 and 10 months, FDG-PET at 13 months revealed that the FDG accumulation of the ex-tumor area had disappeared, and signaling the tumor was controlled. At 40 months, the density of the pneumonitis expanded slightly, and then profoundly at 46 months. Thereafter, the consolidation did not show remarkable changes and the patient was diagnosed as having no recurrence 71 months after SBRT.

**Fig. 10:**
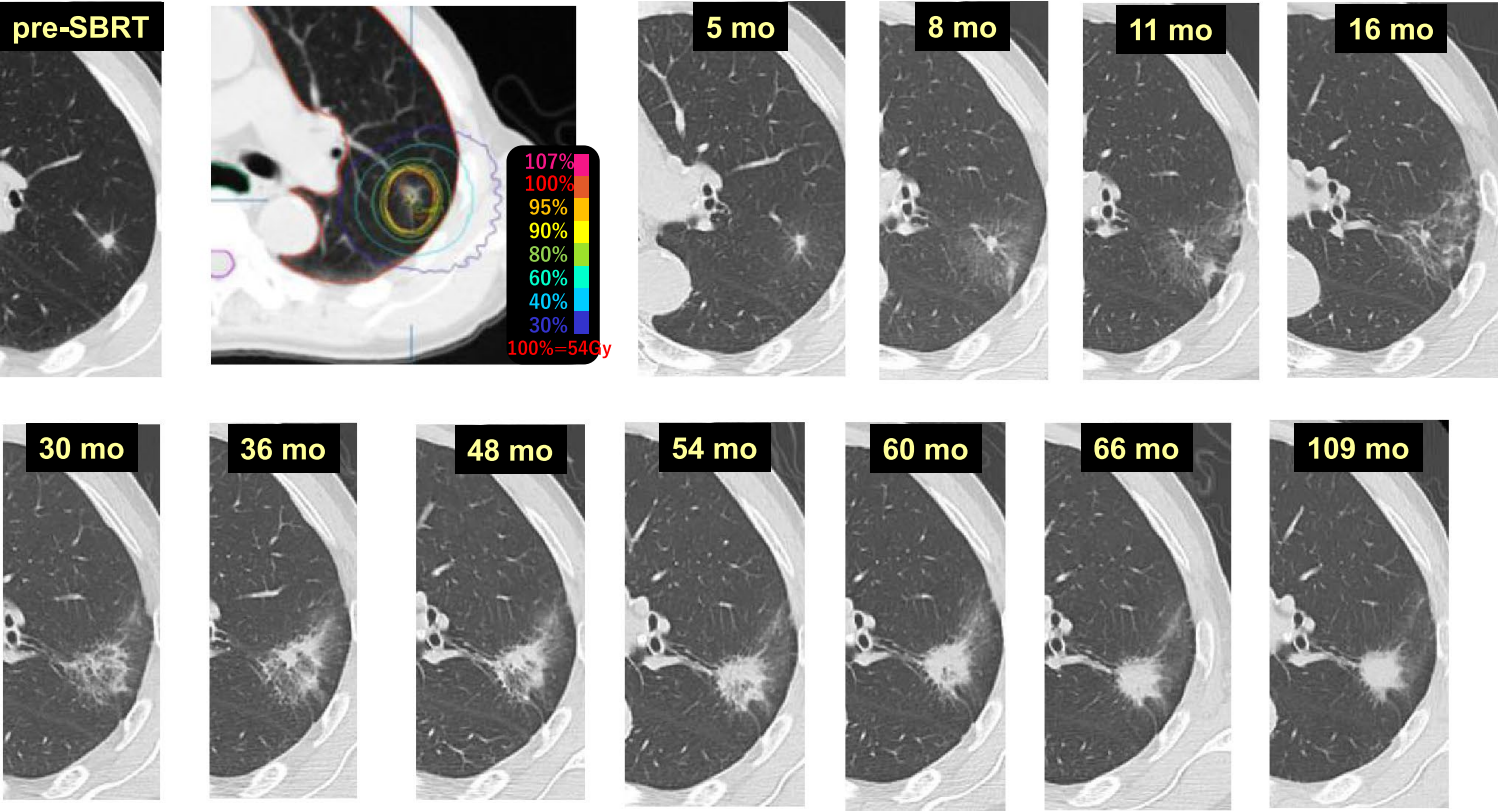
Delayed pulmonary damage (2)

Case 10 (Fig. 10) is a 72-year-old male who received 54 Gy/4 fr SBRT to a lung tumor (histologically unconfirmed) in the left upper lobe (S4). At 5 months after SBRT, no pulmonary changes were observed in the lung area surrounding the tumor, but the density of the target region began to increase slightly after 8 months and continued to gradually increase over time. At 36 months, the density of the target had clearly increased. Thereafter, it slowly converged with an internal area increasing in density in a synchronous manner. The internal air density in the consolidation after SBRT disappeared at 66 months. No remarkable changes were observed at 109 months. This is also a case considered to have recovered from primary lung cancer.

## Increased density of ground glass opacity (GGO)

When administering SBRT to a GGO lesion, CT about 2 months after SBRT reveals an increase and/or expansion of the density of the GGO in many cases. These findings are frequently observed and can be easily diagnosed as “exacerbation” or “relapse” after SBRT to the GGO [11–14]. Tumor edema extending into pulmonary alveoli is a probable cause, rather than simple radiation pneumonitis, because the increased density is mostly limited to GGO in many cases.

**Fig. 11:**
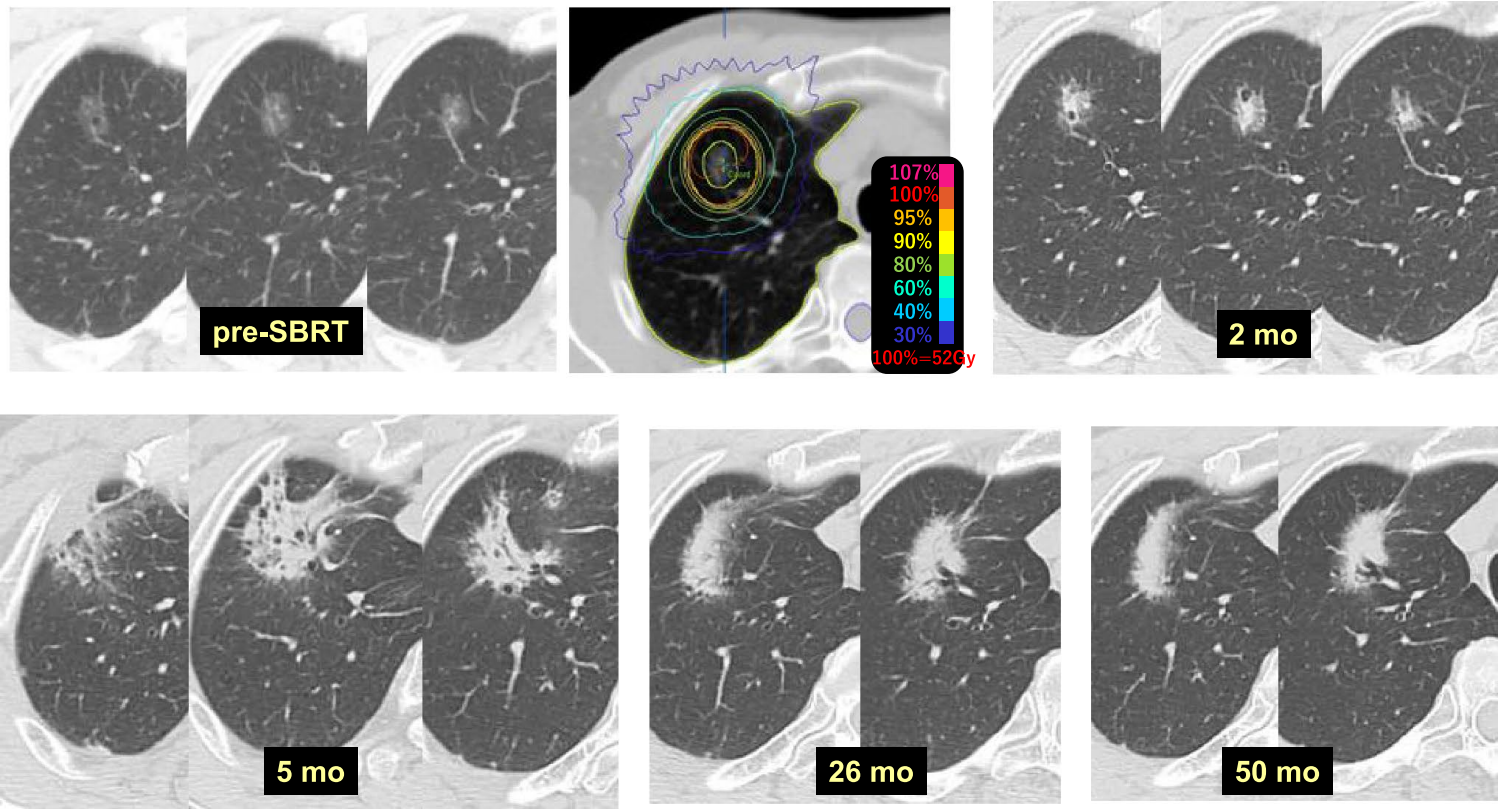
Increased density of ground glass opacity (GGO) (1)

Case 11 (Fig. 11) is a 65-year-old male who received 52 Gy/4 fr SBRT to a GGO lesion in the right upper lobe (S1). Increased density of the GGO was observed at 2 months after SBRT. Radiation pneumonitis appeared at 5 months, and the transition from radiation pneumonitis to fibrosis was thought to be completed at 26 months. No changes in density of any regions due to SBRT were observed at 50 months.

**Fig. 12:**
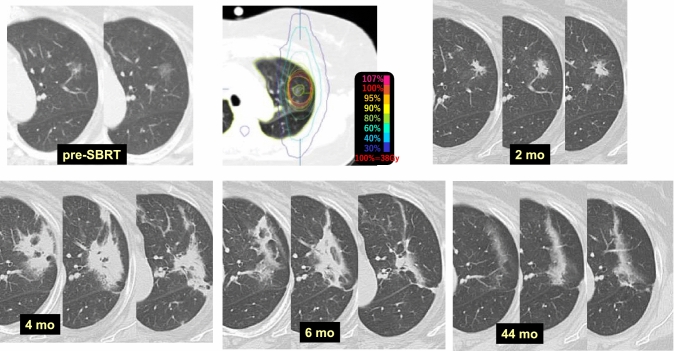
Increased density of ground glass opacity (GGO) (2)

Case 12 (Fig. 12) is a 59-year-old female who received SBRT at D95% = 38 Gy/4 fr (maximum dose, 48.2 Gy) to a GGO in the left upper lobe (S1 + 2). The density of the GGO increased obviously and was also enlarged at 2 months after SBRT. At 4 months, radiation pneumonitis appeared that included the GGO, and evaluation of SBRT efficacy in the tumor was not possible. At 6 months, the radiation pneumonitis appeared to be inactive, and only mild changes indicative of pulmonary fibrosis were observed at 44 months.

## Enlargement of consolidation after SBRT caused by heart failure

Pulmonary fibrosis that progresses from SBRT-induced radiation pneumonitis sometimes worsens with congestive heart failure [12]. We experienced a patient who presented with congestive heart failure clinically, along with enlargement of an SBRT-induced fibrotic consolidation that then decreased in size following a 1-month course of a diuretic agent. Heart failure most likely also affects enlargement of consolidations after SBRT.

**Fig. 13:**
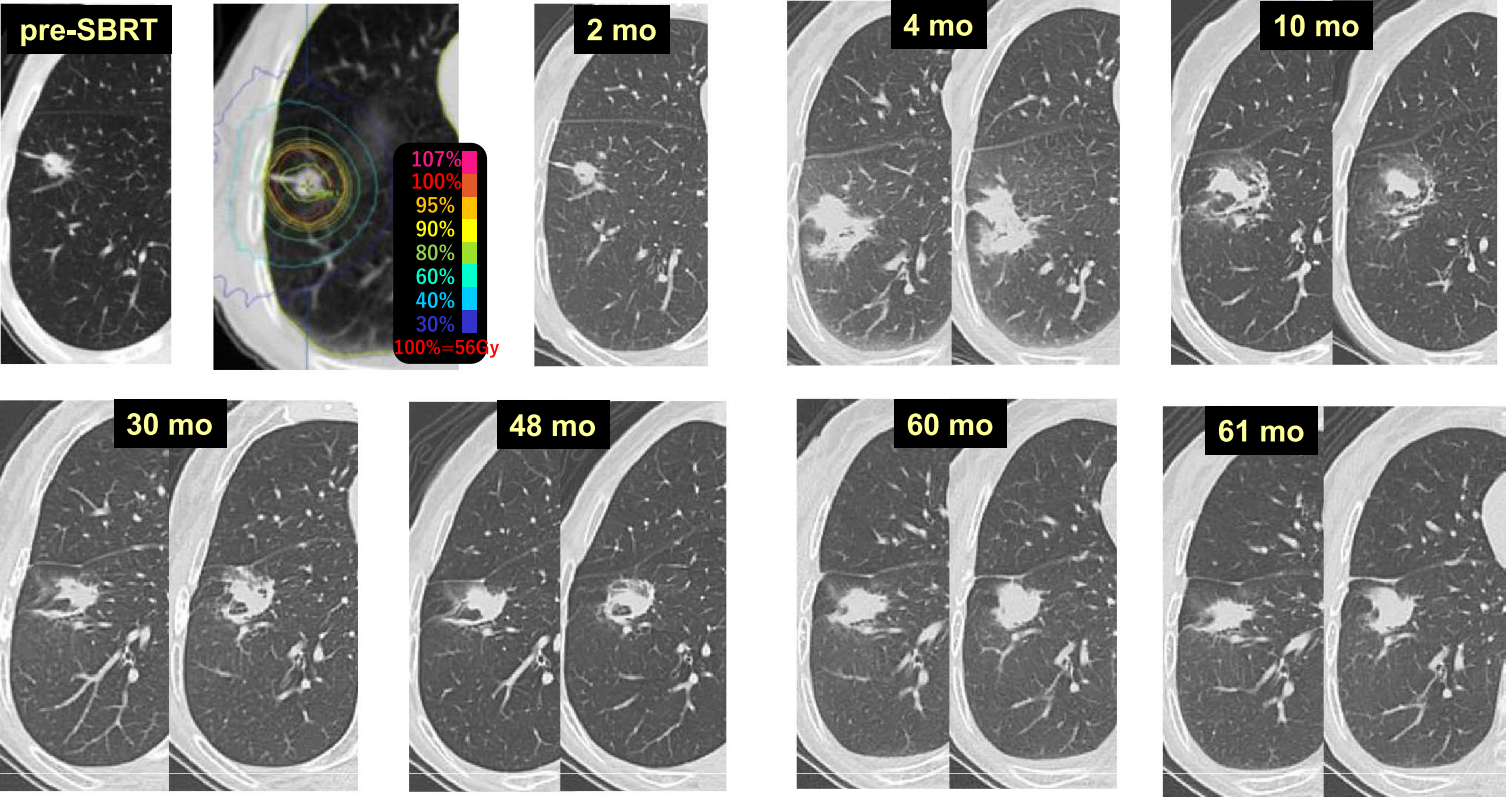
Enlargement of consolidation after SBRT due to heart failure

Case 13 (Fig. 13) is an 83-year-old female who received 56 Gy/4 fr SBRT to a lung tumor (adenocarcinoma) in the right lower lobe (S8). Radiation pneumonitis appeared at 4 months after SBRT and had almost completely improved at 10 months, and no changes in the target lesion were observed for up to 48 months. The consolidation considered to already be fibrotic was enlarged at 60 months. After chronic heart failure was diagnosed due to the presence of dilatation of the heart, bilateral pleural fluid collection, and edema of the lower legs, a diuretic agent was administered for 1 month that improved the heart failure and restored the consolidation to its original size.

**Fig. 14:**
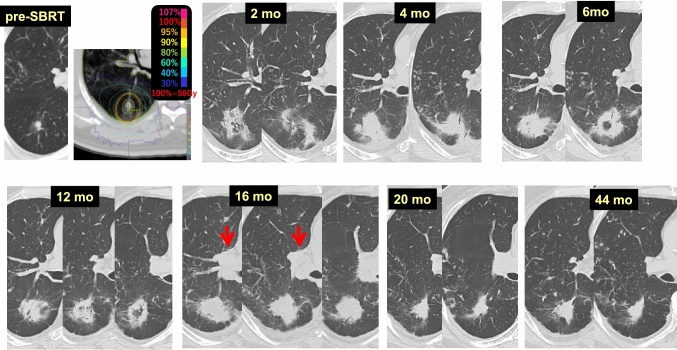
Enlargement of consolidation after SBRT due to hilar and mediastinal lymph-node swelling (± heart failure)

Case 14 (Fig. 14) is an 82-year-old male who received 56 Gy/4 fr SBRT to a lung tumor (adenocarcinoma) in the right lower lobe (S6). Radiation pneumonitis appeared at 2 months after SBRT, and its density had increased along with pleural fluid accumulation at 4 months. The radiation pneumonitis was considered to peak at 6 months, and then, absorption and convergency of the consolidation were considered to continue up to 12 months; however, gradual enlargement of the consolidation was evident at 16 months. Prominent enlargement of the right hilar lymph nodes (red arrows) and right pleural effusion were observed, and systemic lymph-node swelling was also noted at the time. Chemotherapy was administered based on a diagnosis of peripheral T-cell lymphoma. At 20 months, the consolidation was restored to a typical fibrotic lesion. The probable cause of the consolidation enlargement was retention of lymph due to hilar and mediastinal lymph-node lymphoma involvement. However, simultaneous occurrence of congestive heart failure could not be ruled out due to widespread disease. When consolidation enlargement is observed in patients with evidence of heart failure, such as bilateral pleural effusion and cardiac dilatation, tumor recurrence is unlikely and heart failure should be suspected.

## Changes in the thoracic wall and ribs

For treatment of peripheral pulmonary tumors near the chest wall, it is often difficult to control the radiation dose to the thoracic wall and the ribs; therefore, SBRT can cause fibrosis of the thoracic wall soft tissue and fracture of the ribs, which are indicated by mild deformity and bulging of the chest wall. One reported case was suspected to have thoracic wall invasion due to continuity with an intrapulmonary consolidation after SBRT [11, 12, 14]. Chest wall adverse events after SBRT are less common than post-thoracotomy pain syndromes, occurring in about half of all surgical cases [27], and nearly 30% of patients experience pain for 4–5 years [28].

**Fig. 15:**
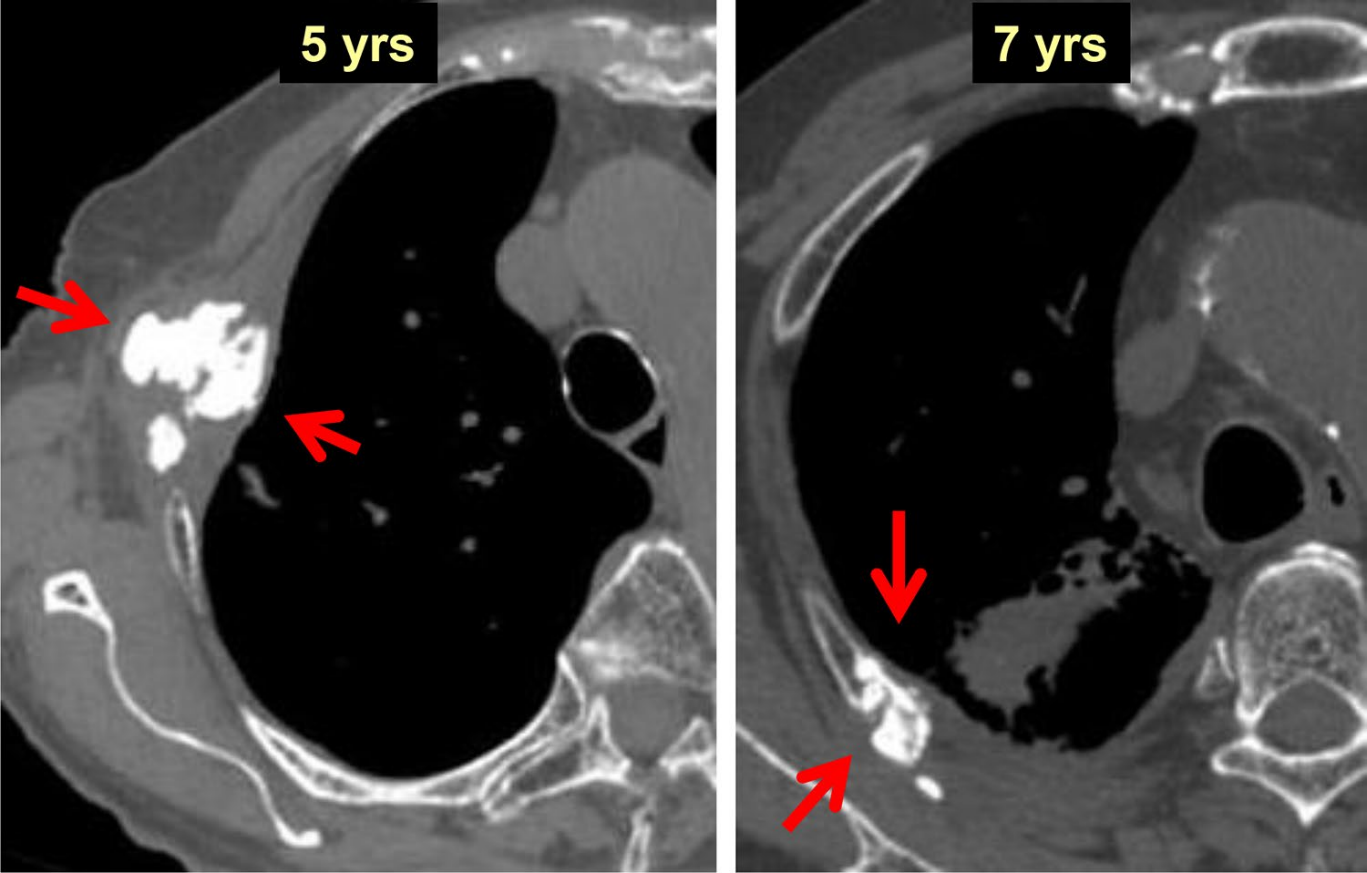
Changes in the thoracic wall and ribs (1)

Figure 15 shows the CT images of rib fractures in patients 5 and 7 years after SBRT. The ribs remained broken without reconnection. Dense calcification was observed in the fractured portion of the ribs (red arrow). In the case 5 years after SBRT, the dense calcification appeared to be spreading around the fracture, partly due to thoracic wall respiratory motions.

**Fig. 16:**
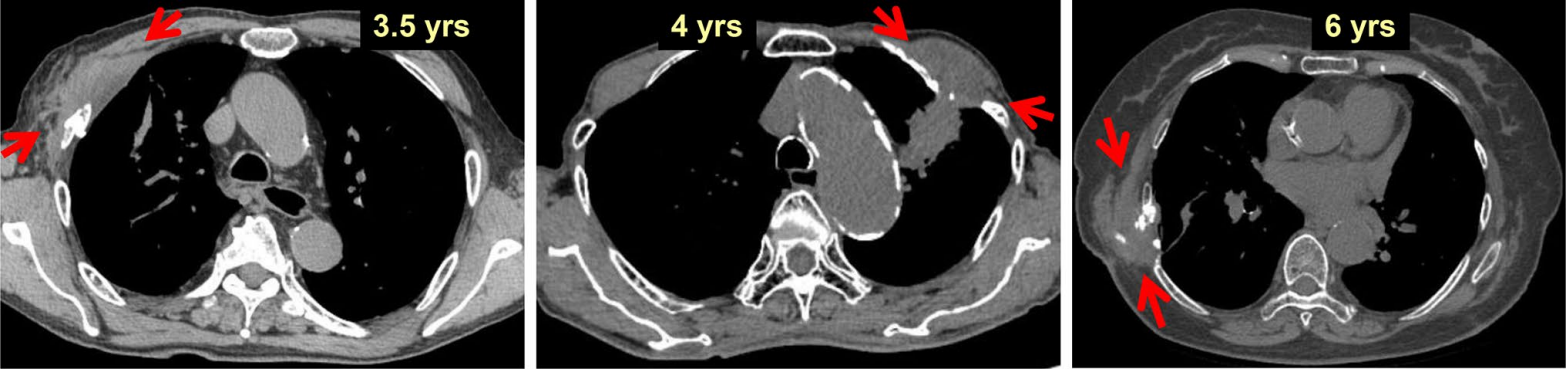
Changes in the thoracic wall and ribs (2)

Figure 16 shows CT images of the chest walls of patients 3.5 years, 4 years, and 6 years after SBRT. Damage to the chest wall appeared as homogenous soft-tissue density similar to that of muscles. These subcutaneous masses demonstrated firmness as if they were ribs. They were considered to indicate fibrosis of subcutaneous thoracic wall soft tissue, including muscles. Rib fractures were almost always observed simultaneously. In all cases shown in Figs. 15, 16, no pain was present at the time of CT. However, mild chest wall pain occurred in some patients during the course of disease.

On bone scintigraphy, the early phase of rib fracture shows band-like accumulation that is specific to cancer metastasis to the rib [14]. Intrapulmonary changes and rib fracture(s) after SBRT are usually observed close to each other in the upper lung lobes due to small respiratory movements in these lobes. In contrast, rib fractures are observed in the chest wall away from the SBRT-treated lesions in the lower lung, because the respiratory phases in SBRT and diagnostic CT scan (deep inhalation) differ markedly in many cases. Although some systematic reviews of the assessment of chest wall toxicity and dose have been published [29, 30], this is unlikely to lead to serious complications, and the dose to the target should be considered with caution.

## False-positive PET results

When SBRT-induced consolidations increase in size during follow-up and tumor recurrence is suspected, FDG-PET may be the first choice to rule this out. Although FDG accumulation is often observed in radiation pneumonitis areas shortly after SBRT, such accumulation is not usually seen after fibrosis is complete. Sometimes, consolidations considered to be completed radiation fibrosis show accumulation of FDG on PET [11, 13].

**Fig. 17:**
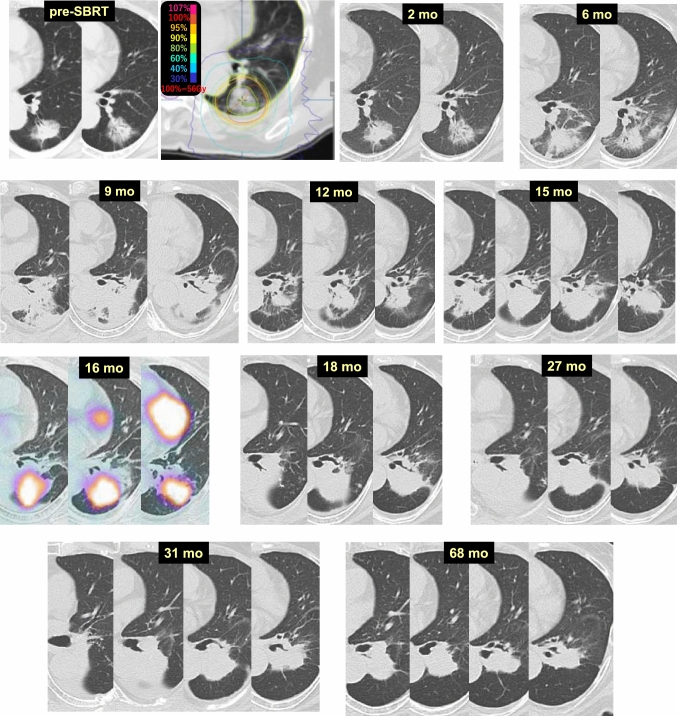
False positive on PET

Case 15 (Fig. 17) is an 83-year-old woman who received 56 Gy/4 fr SBRT to a lung tumor (adenocarcinoma) in the left lower lobe (S6). At 2 months after SBRT, no changes were observed at the tumor site. Radiation pneumonitis appeared 6 months after SBRT, and the peak of pneumonitis occurred at 9 months. Although the radiation pneumonitis was absorbed and converged by 12 months, the consolidation increased at 15 months. Tumor recurrence was suspected at that time, and FDG-PET was performed at 16 months, showing strong FDG accumulation in the consolidation. Bronchoscopy was conducted, but biopsy could not be performed due to SBRT-induced bronchial stenosis. Close follow-up was continued, and the consolidation slowly decreased in size. There was no indication of relapse at 68 months. The FDG accumulation observed on PET was considered to be a false-positive result in this case.

FDG accumulation on PET within 6 months after SBRT for lung tumors is not particularly reliable [31], and some findings suggest that even moderate FDG accumulation on PET at 1 year is not suggestive of recurrence [32].

## Liver parenchymal changes after SBRT

Parenchymal changes in the liver are often observed after SBRT to tumors at the base of the right lung. These changes are sometimes mistaken for liver metastases due to the focal confined hypodensity of the liver [11, 12, 14], called focal liver reaction. This phenomenon appears as localized, well-demarcated radiation hepatitis (hepatopathy).

**Fig. 18:**
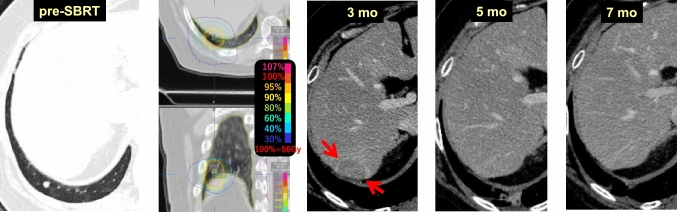
Liver parenchymal changes after SBRT

Case 16 (Fig. 18) is a 72-year-old female who received 56 Gy/4 fr SBRT to a pulmonary metastasis from renal cell carcinoma. At 3 months after SBRT, a hypodense area appeared in the area of the liver parenchyma that had received a high radiation dose (red arrow). At 5 months, the hypodense area (focal liver reaction) was obscured and difficult to see.

The results of SBRT with single-dose irradiation for liver tumors showed sharply demarcated hypodense areas surrounding the treated tumor in 74% of nonenhanced scans [33].

## Conclusion

Radiation pneumonitis after SBRT differs in many ways from that which occurs after conventional radiation therapy in many cases. It is often difficult to accurately diagnose lung lesions after SBRT. Careful judgment is required when determining whether tumor relapse has occurred after SBRT. Comprehensive diagnosis is needed, considering the image course and clinical data. Although the finding of “enlargement” of the consolidation after SBRT for lung tumors is the only clue suggesting recurrence on radiological imaging, it does not always signify a recurrence. Therefore, it is necessary to consider that imaging changes observed in the lung after SBRT may be unique. The best way to achieve the correct diagnosis is to carefully evaluate time-course imaging changes. If recurrence is suspected and surgical options remain, surgery as a diagnostic treatment is recommended before the disease reaches an advanced stage. In cases of no surgical options, even if a relapse is suspected, hasty conclusions should not be made; rather, I recommend CT about 2 months later. Several cases of assumed recurrences were ruled out by follow-up CT, as described herein; I hope that these cases will help you in your daily practice.
